# Supplementary material for: Carbon nanotube-based three-dimensional monolithic optoelectronic integrated system
Source: Nat Commun. 2017 Jun 8;8:15649. doi: 10.1038/ncomms15649 (PMC5472716; doi:10.1038/ncomms15649)
Supplement: Supplementary Information — Supplementary Figures, Supplementary Tables and Supplementary Notes [file ncomms15649-s1.pdf]

Supplementary Note 1

Device fabrication process. Deposition of the high-semiconducting-purity CNTs (Ref 23)——Define test pads and alignment mark patterns (EBL)——Ti/Au (5/40 nm) deposition (EBE)——Define Pd contact patterns (EBL)——Ti/Pd (0.3/60 nm) deposition (EBE)——Define Sc contact patterns (EBL)——Sc (70 nm) deposition (EBE)——Remove those CNTs apart from active channel (RIE)——Define the gate patterns of n-FET and the insulating layer (EBL)——Deposit 20-nm HfO_2 (ALD)—— Deposit 10-nm Pd film as the n-FET top-gate metal (EBE)——Define the emitter contact electrode patterns——Deposit Ti/Au (5/40 nm) (EBE)——In-situ assembling of (8, 3) & (8, 4) CNTs (DEP)——Define the top HfO_2 packaging layer (EBL)——Deposit 20-nm HfO_2 layer (ALD)——Define PMMA cover layer (EBL)

Supplementary Note 2

Calculation of responsivity and detectivity of the cascading detector. The detector operates in photovoltage condition, which can be combined with virtual contacts to increase the signal-to-noise ratio. There are typically three main noise sources in a PV detector, that is, Johnson or thermal noise, which occurs regardless of any applied voltage; shot noise, which may be dominant when only a finite number of carriers are sufficiently small; and Flicker (1/f) noise, which is a resistance fluctuation and is always related to a direct current. In our case, there is no direct current flowing in the conducting channel because of the open-circuit operation mode. In principle, there is no shot noise, and the 1/f noise will not be dominant. Therefore, the Johnson noise will be the dominant factor in this condition. Above results have been discussed previously[23]. The photovoltage responsivity is defined as follows:

$$R_v = \frac{v_s}{P_{in}}$$

where v_s is the signal voltage and P_{in} is the practical incident power on the active area of the detector.

The detectivity $D*$ is defined as follows:

$$D^* = \frac{R_v A^{1/2}}{v_n}$$

where A is the active area of the detector and v_n is the noise voltage.

In this experiment, the signal voltage of the detector is approximately 10 mV under an incident illumination density of 1.45×10^{-5} W/cm^2 at 1,800 nm. Therefore, the responsivity is calculated as approximately 10^8 V/W. The typical noise under a zero bias condition is approximately 2.57×10^{-6} V/Hz$^{1/2}$. Therefore, we calculate that the detectivity is approximately 10^{11} Jones.

Supplementary Note 3

Low-temperature fabrication process. In this 3D OEIC, the bottom-layer functional cells were fabricated via doping-free method, in which the highest temperature of the process is 170 °C (corresponding to the highest PMMA bake temperature) and is considerably lower than the silicon-circuit damage temperature of 400 °C. The top-layer devices were fabricated on an HfO_2 insulator, which was deposited via a low-temperature ALD process (90 °C). The CNT operation layer can be peeled off with the high-temperature CNT growth process of > 800 °C, enabling construct multilayer 3D OEICs. Here, we select the DEP method to assemble top-layer CNTs[30], which is carried out at room temperature (25 °C). Therefore, the highest temperature in the fabrication process of the 3D OEIC is 170 °C, satisfying the back-end-of-line standards for multilayer construction of OEICs.

Supplementary Note 4

Estimation of the emission energy. The measurement system is shown in Fig. S8, consisting of a 50× Leica microscope, a series of lens and reflectors, a confocal pinhole, a slot, gratings and a spectrometer. Reflector A is not used during the practical measurement of the electrically-driven IR emission. Therefore, the efficiency of the system can be written as follows:

$$\eta = \eta_{lens} \cdot \eta_{lens2} \cdot \eta_{trans} \cdot \eta_{\det ector}$$

$$\eta_{lens} = \frac{3}{4\pi}\int_0^{2\pi} d\phi \int_0^{\arcsin(NA/n)} d\theta \sin\theta = \frac{3}{2}\left(1-\sqrt{1-(NA/n)^2}\right)$$

where η_{lens} is the efficiency of the microscope. NA is the numerical aperture, with a value of 0.55. n is the refractive index of the coated passivation PMMA layer, with a value of 1.49. Therefore, the efficiency of microscope can be calculated as approximately 10.6%.

η_{lens2} is the transmissivity of a series of lenses and reflectors. According to the reciprocity principle of light, the efficiency can be achieved by comparing the laser power before and after transmitting through these lens and reflectors. The laser ray is depicted with green lines. Assuming that all of the illumination can reach the sample due to the high collimation characteristics of the laser, the transmission efficiency was determined to be approximately 55.8% after separately measuring the power under the microscope and behind reflector A.

η_{trans} is the transmissivity of a series of lenses between reflector A and the final detector. Part of the laser energy can pass through reflector A and enter the final detector because of the high energy of the laser. Therefore, the energy after reflector A can be measured and regarded as P_3, and the relation between P_3 and the measured counts of the detector can be written as follows:

$$\eta_{\det ector} \cdot \eta_{trans} \cdot P_3 = \frac{\alpha \cdot counts \cdot \hbar\omega}{t}$$

in which $\eta_{detector} \cong 85\%$, $\alpha = 1545e^{-}/count$, and the wavelength of the laser is 1,300 nm. We can achieve $\eta_{trans} \cong 4.74\ \%$.

Then, we determine that $\eta \cong 2.38 \times 10^{-3}$.

The external efficiency of the device can be written as follows:

$$\eta_{EL} = \frac{\alpha \cdot counts / \eta}{I \cdot t / 2q}$$

Counts are the sum in the detectable range, I is the device current, and t = 90 s; therefore,

$$\eta_{EL} \approx 2.3 \times 10^{-9} \times \frac{counts}{I(\mu A)}$$

This formula is for an ambipolar device, in which both electrons and holes contribute to the final current; thus, the total number of electron-hole pairs of $I \times t/2q$, in which q is the elementary charge of $q \cong 1.6 \times 10^{-19}$ c.

The final illumination power of the emitter can be written as $P_{EL} = \eta_{EL} \times P_{electrical}$.

Supplementary Note 5

Analysis of the system response speed. The response is limited at ~kHz level (Fig. 1i), which can be analyzed by considering the parasitic capacitances. Therefore, we refer the results shown in Ref (Zhang, P. P. et al, 2015, Nano Res., 8(3), 1005), in which corresponding analyses of capacitances dominated response have been discussed. It is clearly demonstrated that the working frequency will be lagged at ~kHz level when the system capacitance dominated. The lumped capacity C_{sys} consists of parasitic capacitances from probes, coaxial cables and measurement instruments. The delay of the system was measured about 40 ns by shortening the two probes directly and the system resistance R_{sys} is ~5 Ω according to the measured results. Therefore, the C_{sys} can be defined as 8×10^{-10} F ($\tau=R_{sys}\times C_{sys}$). When involving this large capacitance, the system response speed is

restricted at ~kHz level (as shown in Table 3 of Ref Zhang, P. P. et al, 2015, Nano Res., 8(3), 1005). More detailed simulation results have also been shown in the reference, which agrees with corresponding experimental ~kHz response speed.

Supplementary Note 6

Patterns/Layers alignment process. The patterns/layers are aligned using 3-points alignment marks as shown in Fig. S5a (highlighted using black squares). When we fabricate the system, all the structures are fabricated according to these three manual marks, enabling them locate on the correct position for alignment. In detail, we take the cascading Pd-Sc contact electrodes as an example to illustrate this process. In Fig. S5b, the Pd electrode patterns can be defined using the alignment marks via EBL, then using EBE to evaporate Pd film. Secondly, we repeated above processes to define the Sc contact patterns and evaporate Sc film (Fig. S5c). Similarly, if all the fabrication processes are carried out according to these same 3-points marks, then patterns/layers can be well aligned.

Supplementary Note 7

Comparisons between the performance of the single layer devices and how they change once they are stacked together. As shown in Fig. S7a, the performance of the CNT transmitter is nearly the same before and after stacking. Then we measured the performance of individual CNT based cascading detector as shown in Fig. S7b. After stacking, the performance of the integrated system has already been shown in Fig. 4d. The photovoltage of the CNT-based nine-cell cascading detector is about 82 mV under illumination power of 5.92 μW (similar to the radiated power of the CNT emitter). As a comparison, the photovoltage after stacking is 55 mV (shown in Fig. 4d). We

contrast the differences of the two light sources condition, i.e., external continuous laser and on-chip CNT emitter. In the stacked devices, the CNT emitter is located in the middle of the active detector channel with effective length of 0.5 μm. Therefore, only the middle of the detector channel is effectively illuminated by the CNT emitter as shown in Fig. 4b and inset of Fig. 4d. However, when we test the individual CNT detector, the external light sources illuminate the entire channel as shown in the inset of Fig. S7b. Experimentally, we designed such an experiment, in which we used 100-nm Ti photon isolation layer to cover the regions where were not illuminated by the emitter in the stacked devices shown in the inset of Fig. S7c. The photovoltage of the detector became 48 mV (Fig. S7c), which is similar to the value after stacking.

Supplementary Note 8

FEM simulation process. To estimate the power from the top-layer CNT emitter on the bottom-layer detector, we simulated the radiation distribution using the finite element method. The two-dimensional simulated region is surrounded by the perfectly matched layer (PML). The thickness of SiO_2, HfO_2 insulating layer and Au electrodes are 500 nm, 20 nm, 40 nm, separately. An electric dipole is utilized as the light source because CNTs are dipole-allowed emission (Refs 31, 32). Corresponding permittivity of materials in the simulations were set according to the data in the literature (Palik, E. D. Handbook of Optical Constants of Solids II; Academic Press: Orlando, 1985).

Supplementary Note 9

Long-time illumination stability of the logic device. We want to prove that the emitted photons from the CNT emitter will not affect the operation of the FET-based CNT logic devices. For further

convincing this goal, we utilized a p-FET under the illumination power of 1.19 μW and 5.92 μW (these powers are similar to that emitted by the CNT transmitter) for ten minutes separately, and then compare its transfer characteristics with the original behaviour. As shown in Fig. S11b, there is not any obvious change of the p-FET transfer curve including the on/off-state current magnitude and threshold voltage. Thus, we utilized both n-FET (Fig. S11a) and p-FET (Figure S11b) to prove that the emitted photons from the emitter will not affect the performance of the CNTs-based logic devices.

Supplementary Note 10

How to avoid the interference between emitters during the mapping process. The interference between emitters during the mapping process can be avoided by using photon isolation layer. As shown in Fig. S12, the CNT emitter can be covered by 100-nm Ti as the photon isolation layer. The calculated photon transmission probability of the 100-nm Ti is on the order of $\sim 10^{-2}$ (inset of Fig. S12b), giving rise to low transmission. Thus, as compared with Fig. S12a, there is not any emitted IR light can be detected as shown in Fig. S12b.

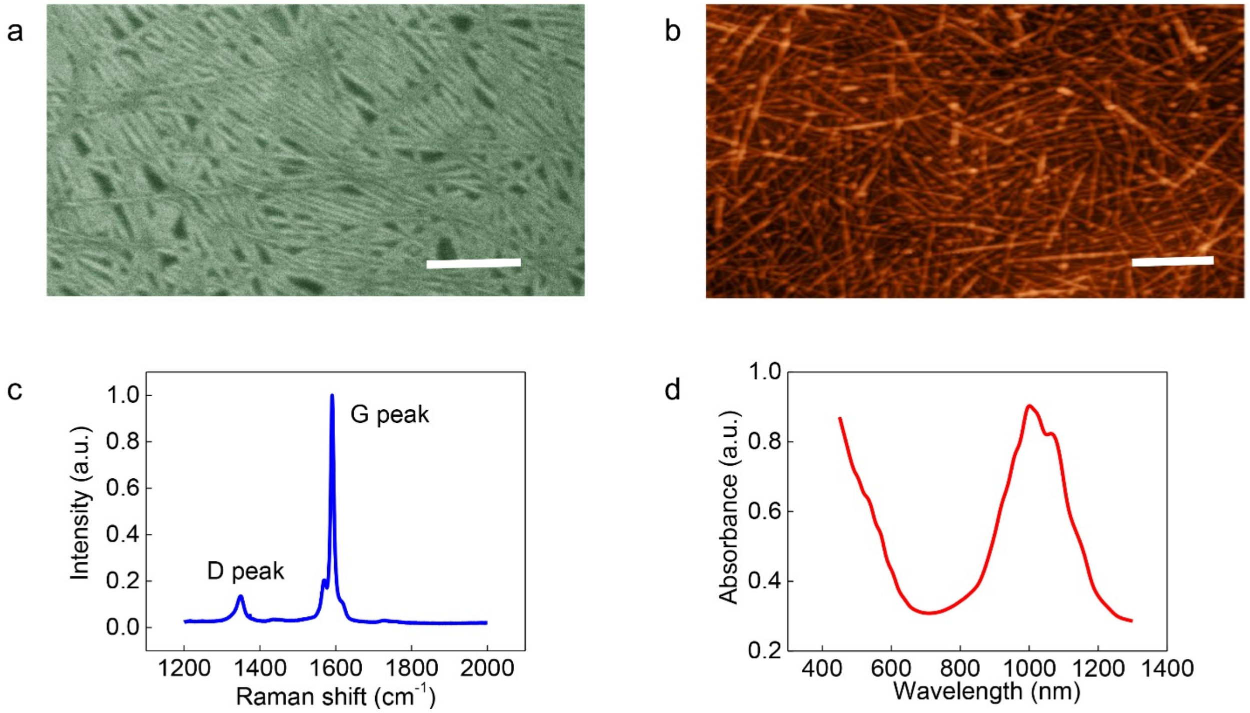

Supplementary Figure 1 | Characterization of high-semiconducting-purity carbon nanotube network film. a, SEM (scale bar, 200 nm) **b,** AFM (scale bar, 200 nm). **c,** Raman spectrum. **d,** Absorbance spectrum.

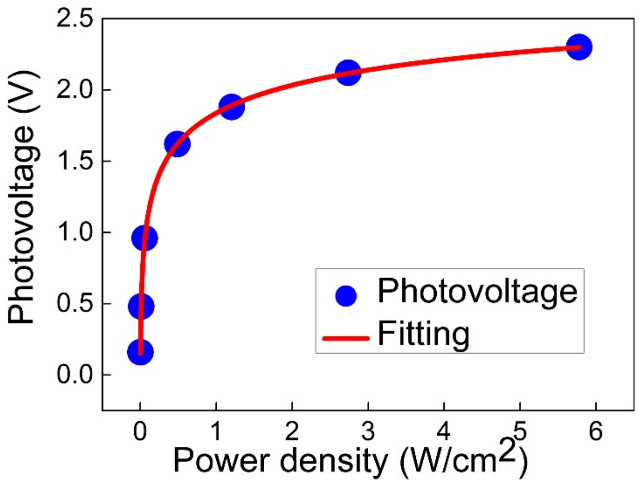

Supplementary Figure 2 | The photovoltaic behaviour of the cascading detector.

	Photocurrent responsivity	Photovoltage responsivity	Electrical on/off ratio	Optical on/off ratio
Ref 29	1 A/W	1×10^5 V/W	10^5	10^4
This work	0.67 A/W	1×10^8 V/W	10^5	10^5

Supplementary Table 1 | Contrast of the photocurrent/photovoltage responsivity, electrical and optical on/off ratio of the device with the past reports.

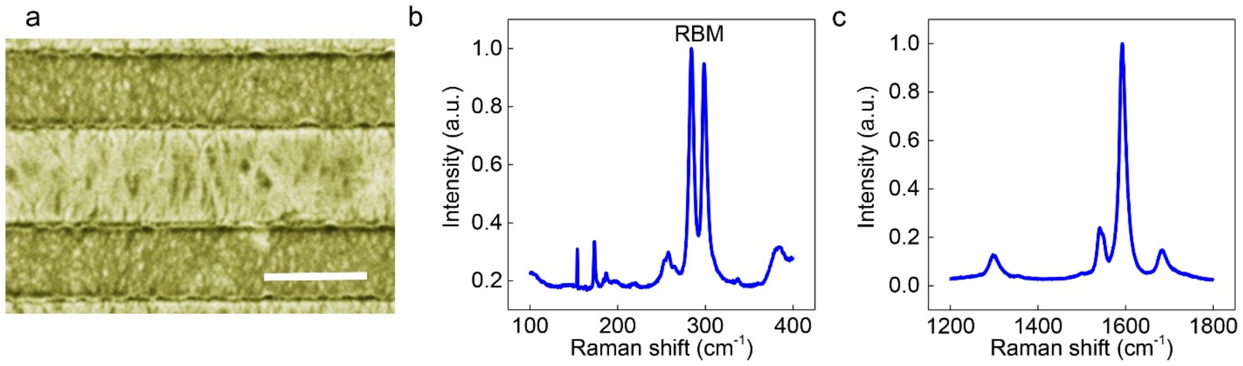

Supplementary Figure 3 | Characterization of (8, 3) and (8, 4) carbon nanotubes. a, Morphology of ac dielectrophoresis fabricated carbon nanotubes (scale bar, 400 nm). **b,** Raman RBM peak. **c,** Raman D and G peaks.

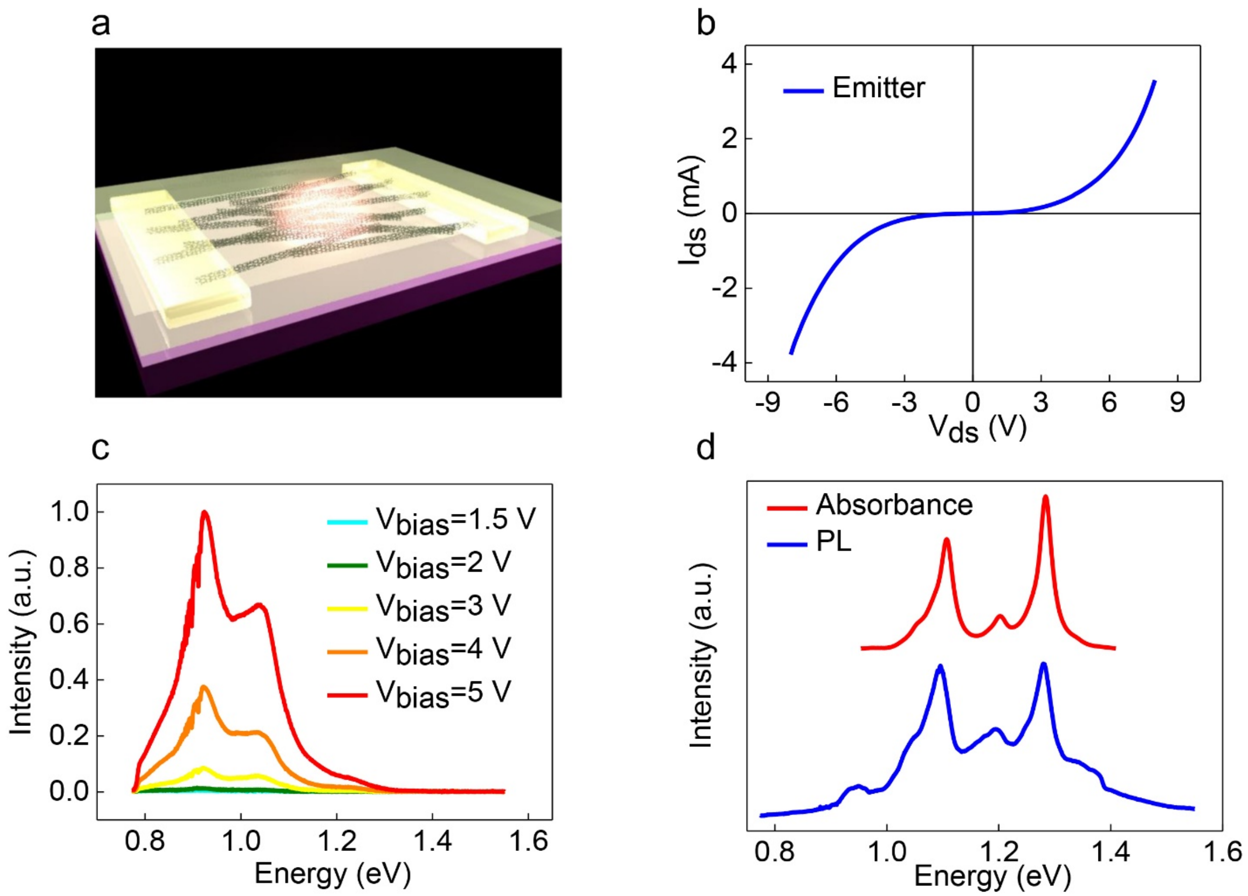

Supplementary Figure 4 | Characteristics of the CNT emitter. a, Schematic structure of the emitter. **b,** Output characteristics. **c,** EL spectra with different voltage biases. **d,** Absorbance and PL spectra of (8, 3) and (8, 4) CNTs.

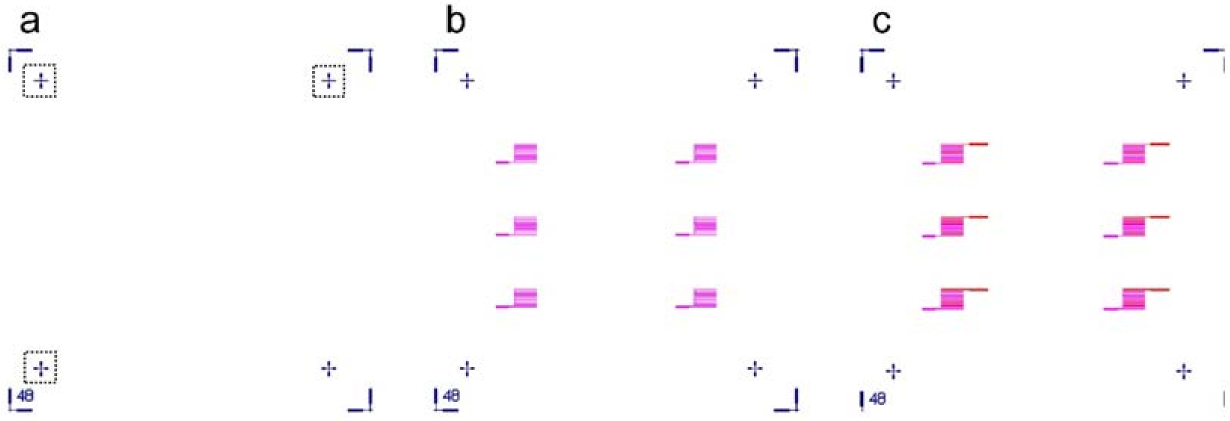

Supplementary Figure 5 ｜a-c, Aligning process of the cascading detector using 3-points alignment marks.

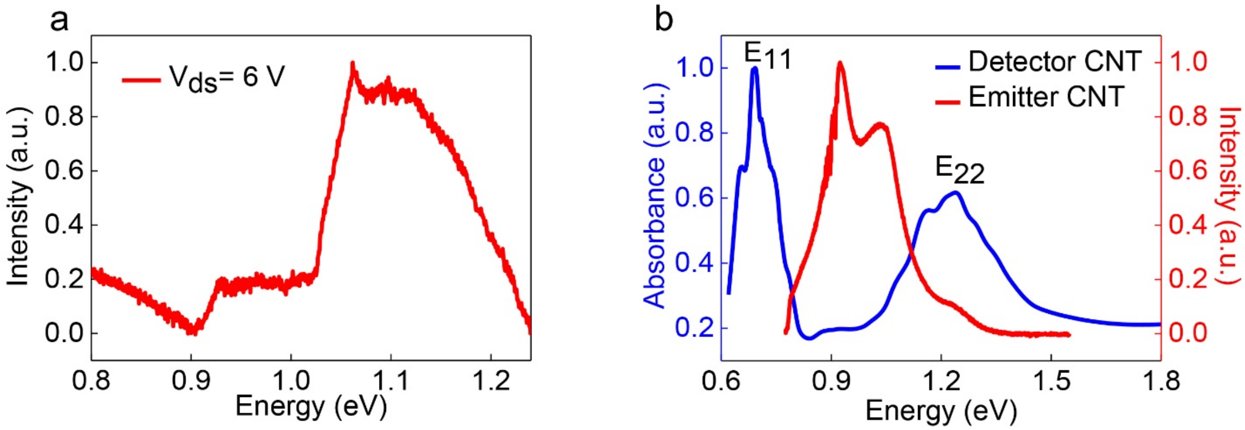

Supplementary Figure 6 ｜EL spectrum of a general semiconducting CNT thin film without specific chirality (a). Typical EL spectrum of the top-layer emitter and the absorbance spectrum of the bottom-layer photodetector (b).

	PMMA bake	ALD	DEP	ICP	EBL	EBE
Temperature(℃)	170	90	25	25	25	25

Supplementary Table 2 ｜Summary of temperatures involved in the fabrication of CNT OEIC.

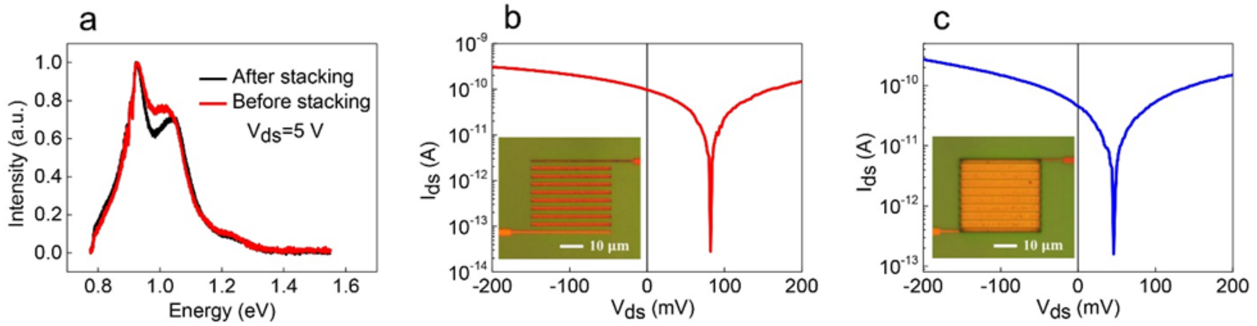

Supplementary Figure 7 | a, The emission characteristics of the CNT emitter before and after stacking. b, Photovoltaic response of the CNT detector. Inset is the corresponding optical image. c, Photovoltaic response of the CNT detector with 100-nm Ti to cover the regions where were not illuminated by the CNT emitter. Inset is the corresponding optical image.

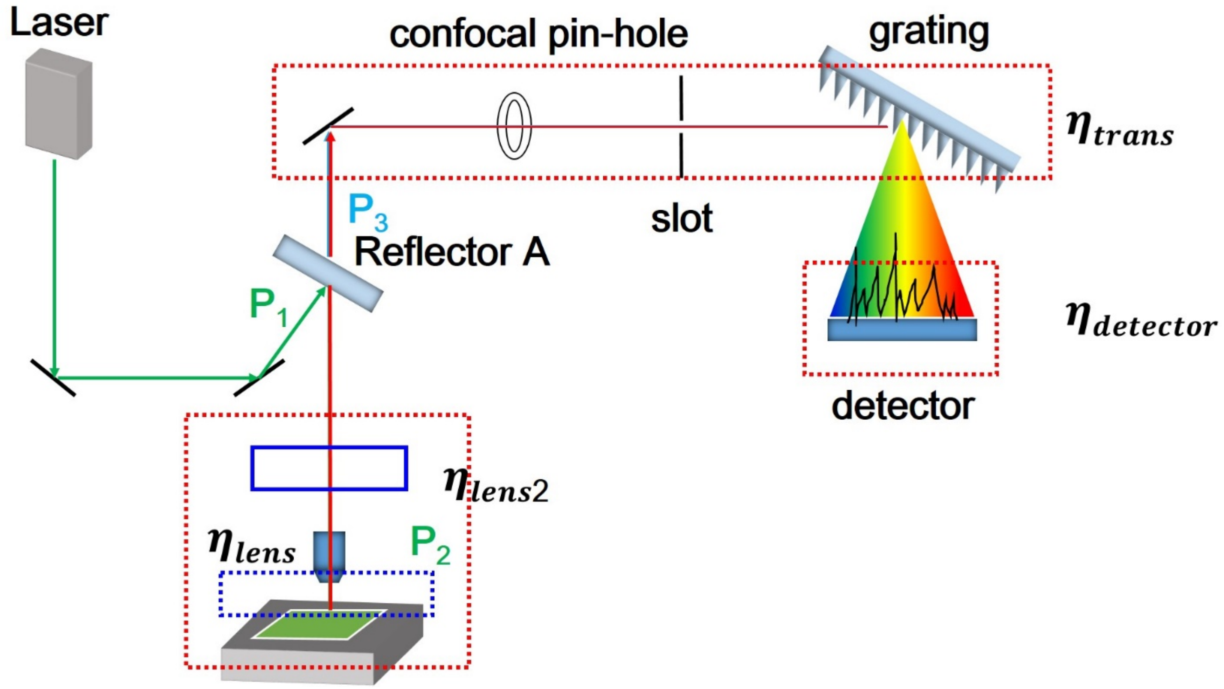

Supplementary Figure 8 | Illumination power calibration system.

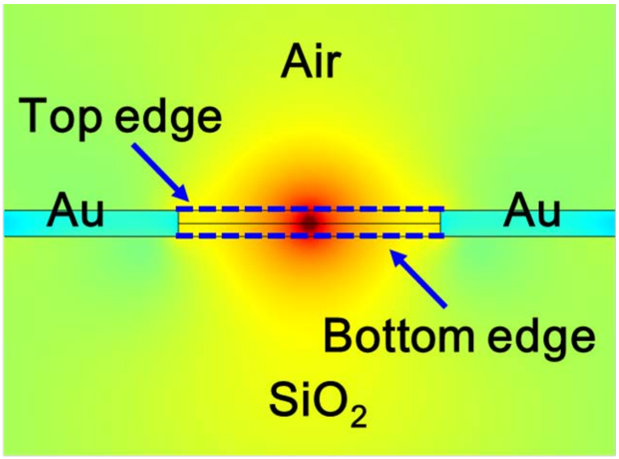

Supplementary Figure 9 | The simulated emission energy distribution of the top-layer CNT emitter.

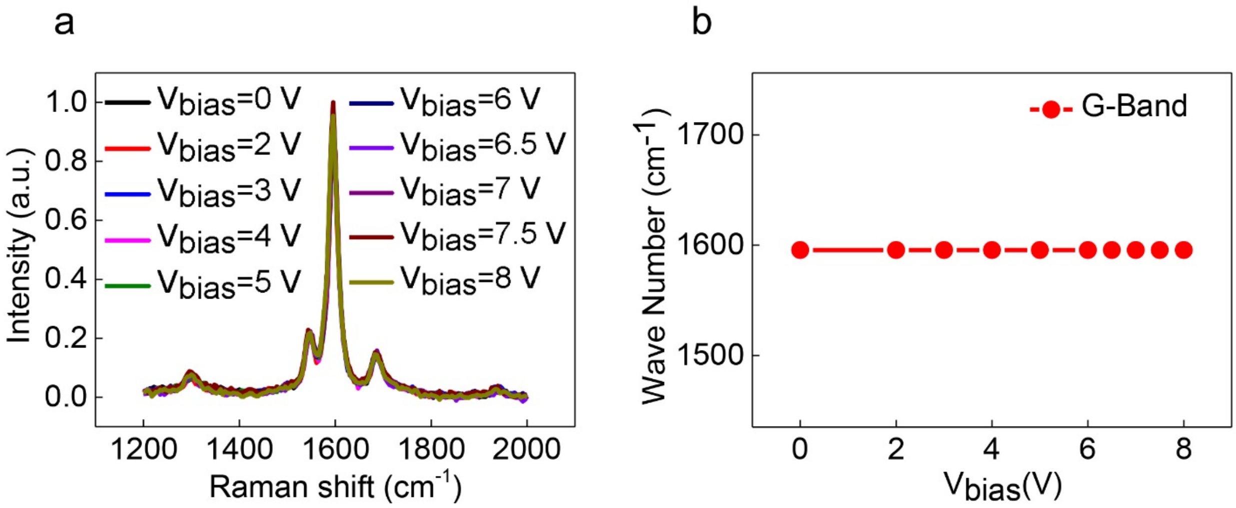

Supplementary Figure 10 | Real-time Raman characterization of the emitter. a, D and G peaks with different voltage biases. **b,** Peak position statistics with different voltage biases.

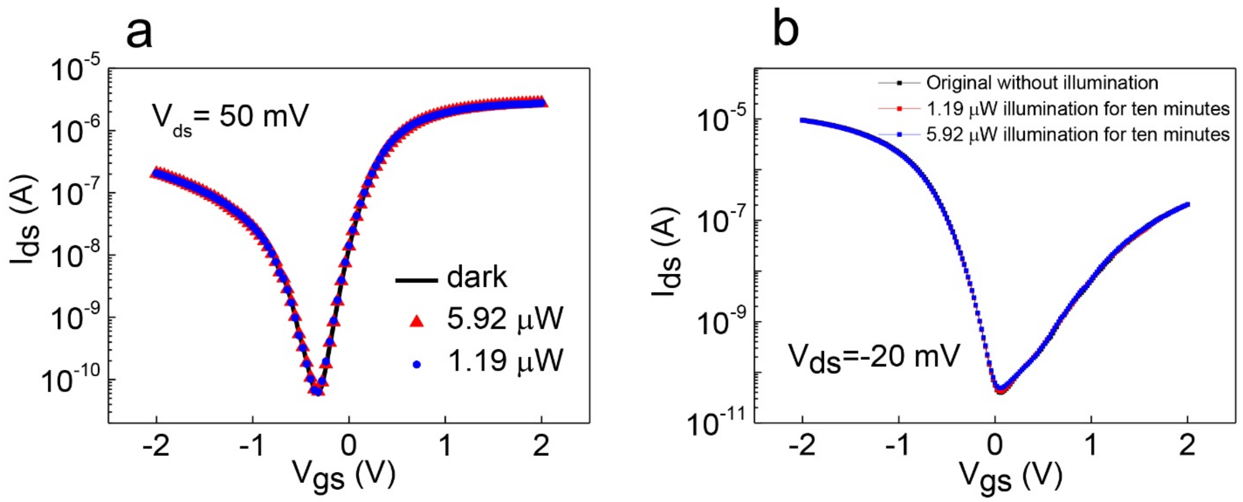

Supplementary Figure 11 | a, Transfer characteristics of CNT n-FET with or without IR illumination. b, Long-time illumination stability of the p-FET.

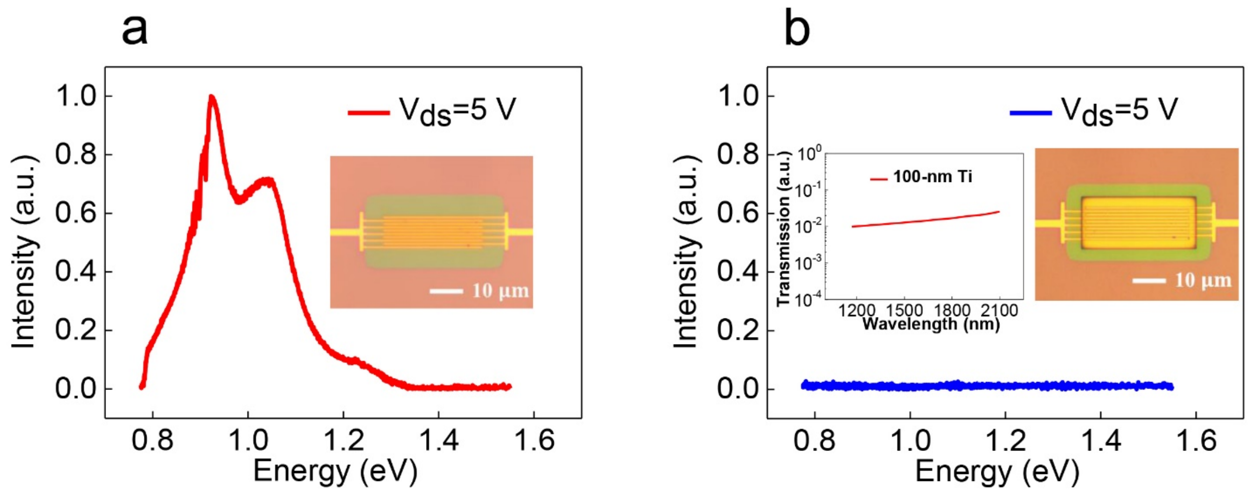

Supplementary Figure 12 | a, EL emission characteristics of the CNT emitter. Inset is the optical image. b, Corresponding emission behaviour with 100-nm Ti covered on top. Inset: Left: Calculated photon transmission probability of 100-nm Ti photon isolation layer. Right: Corresponding device optical image.

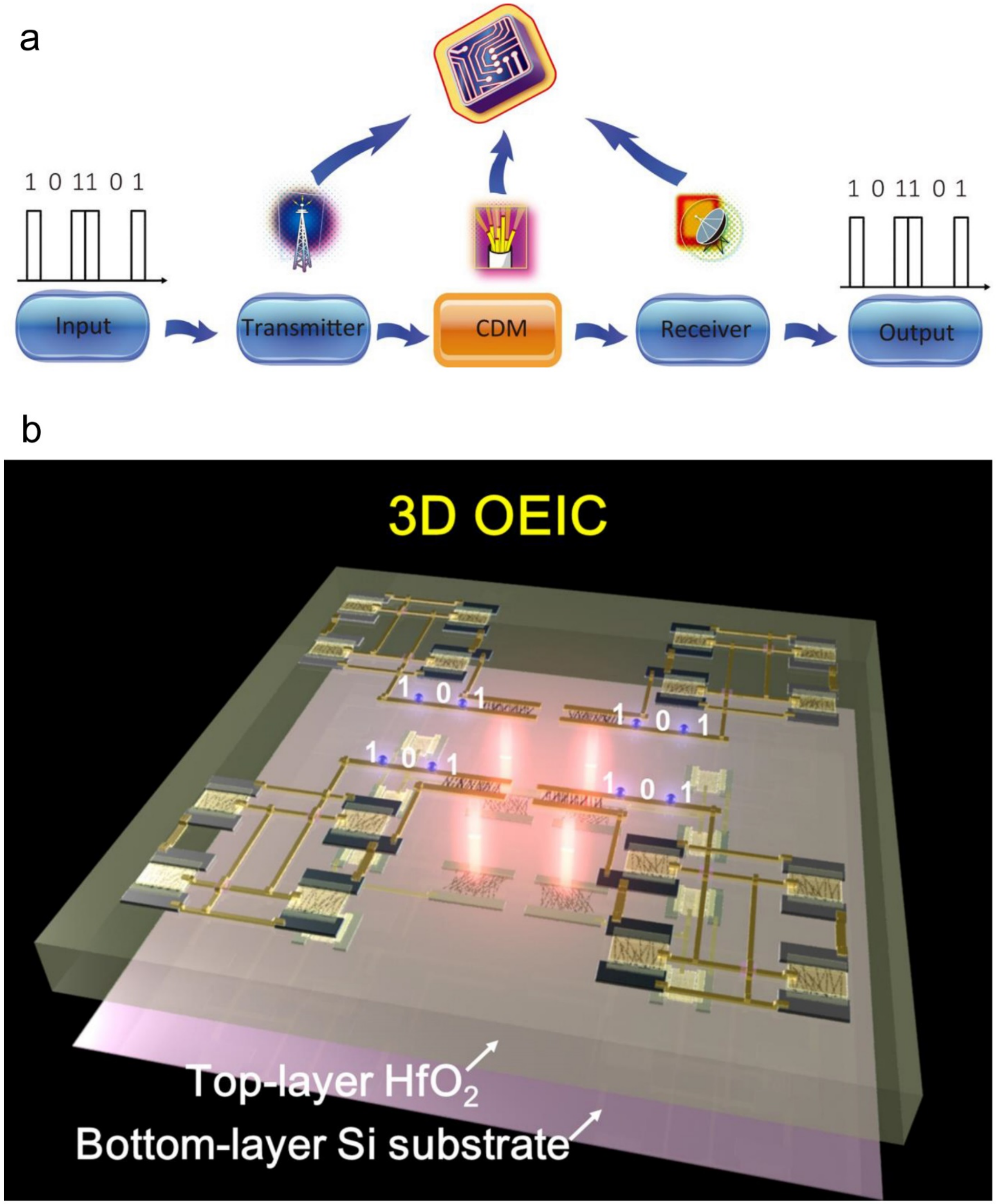

Supplementary Figure 13 | Monolithic 3D OEIC system. a, Schematic of the monolithic binary on-off keying digital OEIC system communicated via CDM. **b,** Schematic of the CNT-

based vertical parallel-transmission OEIC consisting of two layers, that is, the top-layer memory and transmitter; the bottom-layer CPU and receiver. The stored electronic information can be transmitted in parallel via a transmitter/receiver array using light as the information carrier.
